# Dihydropyranocoumarins Exerted Anti-Obesity Activity In Vivo and its Activity Was Enhanced by Nanoparticulation with Polylactic-Co-Glycolic Acid

**DOI:** 10.3390/nu11123053

**Published:** 2019-12-13

**Authors:** Abu Yousuf Hossin, Masashi Inafuku, Hirosuke Oku

**Affiliations:** 1 The United Graduate School of Agricultural Sciences, Kagoshima University, Kagoshima 890-0065, Japan; yousuf.uoda@gmail.com (A.Y.H.); okuhiros@comb.u-ryukyu.ac.jp (H.O.); 2 Tropical Biosphere Research Center, University of the Ryukyus, Senbaru 1, Nishihara, Okinawa 903-0213, Japan; 3 Faculty of Agriculture, University of the Ryukyus, Senbaru 1, Nishihara, Okinawa 903-0213, Japan

**Keywords:** *Peucedanum japonicum* Thunb, dihydropyranocoumarins, anti-obesity, high-fat diet-induced obesity, PLGA nanoparticle

## Abstract

Dihydropyranocoumarins (DPCs) were isolated from *Peucedanum japonicum* Thunb as anti-obesity compounds in 3T3-L1 adipocytes assay; however, it is uncertain whether DPC exerts anti-obesity activity in vivo. Therefore, this study evaluated the oral intake of pure DPCs in mice fed a high-fat diet, and also attempted to enhance its activity by nanoparticulation. Increases in body weight gain and fat accumulation in white adipose tissues were significantly suppressed by the dietary intake of DPCs (1.943 mg/mouse/day). DPCs intake also significantly decreased the mean size of adipocytes and upregulated mRNA levels of thermogenesis-related genes. Nanoparticulation of DPCs with polylactic-co-glycolic acid (PLGA) dramatically increased its activity almost 100-fold over that of a non-nanoparticulated form. Thus, our findings clearly demonstrated the anti-obesity activity of DPCs in vivo and suggested that PLGA nanoparticle encapsulation was useful to enhance the anti-obesity activity of DPCs with the aim to develop natural and safe anti-obesity agents.

## 1. Introduction

Obesity is well known as a major global health issue and several therapeutic agents have been developed for treating this condition by reducing nutrient absorption or by enhancing thermogenesis and lipid turnover [1,2]; however, considering the safety of these agents and their adverse side effects, medicinal plants and their active compounds merit investigation for the development of more natural and safer anti-obesity agents.

The nutritional value of food can be improved by adding bioactive compounds; however, most of their positive effects are impaired by their poor bioavailability, limited water solubility, and metabolic transformations [3]. One strategy for reducing these limitations is to integrate these bioactive compounds into nanoparticles. Delivery systems using nanoparticles have been investigated as a possible approach to markedly improve the bioavailability of drug and food bioactives [3,4]. Carrier systems of biodegradable particulates are of interest as a potential means to orally delivery compounds to improve their bioavailability [5]. Among the various polymers, polylactic-co-glycolic acid (PLGA) has received the most attention because of its favorable degradation characteristics and biocompatibility and has been approved by the United Stated Food and Drug Administration and European Medicine Agency for drug delivery use [6,7,8].

Several studies have reported that *Peucedanum* has various pharmacological properties [9,10,11], and several types of coumarin and essential bioactive substances have been isolated from these plants [12,13]. *Peucedanum japonicum* Thunb (PJT) is a wild herbal plant that grows along the ocean cliff areas throughout southern Japan and China. The leaves of this plant have been used as a leafy vegetable and garnish and are well-known as “Choumeisou” in Japanese, which denotes a long-life herb in Okinawa, Japan. The results of our previous studies [14,15,16] have suggested that dietary intake of PJT ameliorates obesity and diabetes symptoms in mice fed a high-fat diet (HFD) and revealed that several types of dihydropyranocoumarins (DPCs) from PJT have inhibitory effects on lipid accumulation of differentiated 3T3-L1 adipocytes. We have also shown that dietary intake of DPC concentrate (purity ~60%) prepared from PJT significantly decreases the relative weight of white adipose tissue (WAT) in HFD-fed mice; therefore, we suggested that DPCs could be an anti-obesity compound of PJT [14], although these findings have not excluded the possibility that other impurities in DPC concentrate also have anti-obesity effects. Thus, the aim of this study was to elucidate the anti-obesity effects of DPCs using purified DPCs on HFD-induced obesity in mice. As part of our study, we also assessed the effect of nanoparticulation of DPCs into PLGA to maximize their bioavailability and functionality.

## 2. Materials and Methods

### 2.1. Purification of DPCs from PJT and Preparation of Nano-DPCs

**Figure 1:**
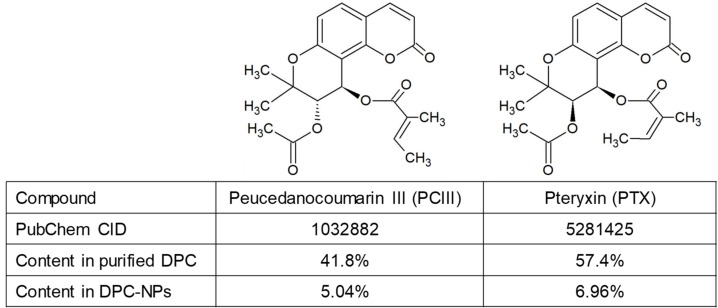
Chemical information and content of dihydropyranocoumarins (DPCs) used in this study. Notes: DPC, dihydropyranocoumarin and nano-DPC, DPC-encapsulated polylactic-co-glycolic acid nanoparticles.

The PJT leaves used in this study were cultivated and harvested on Yonaguni, an island in Okinawa Prefecture, Japan. DPC concentrate was prepared from the dried PJT leaves according to the methods used in our previous study [14]. The primary concentrate was further concentrated using a Hi-Flash column ODS-MS (YANAZAN Corp., Osaka, Japan), and subsequently evaporated. To purify DPC, the concentrates were applied onto an Inertsil ODS-3 column (20 × 250 mm, GL Science Inc., Tokyo, Japan). The DPC fraction was collected, evaporated, and its purity was determined using a COSMOSIL 2.5Cholester column (2.0 × 100 mm, Nacalai Tesque, Inc., Kyoto, Japan) on the Shimadzu LC-20A high performance liquid chromatography (HPLC) system (Shimadzu Corp., Kyoto, Japan). Isolated DPCs were also nanoparticulated with PLGA as nano-DPCs. The Vehicle PLGA nanoparticles and nano-DPCs used in this study were prepared by SENTAN Pharma Inc. (Fukuoka, Japan), and the particle mean size were approximately 304 nm and 266 nm, respectively (Figure 1). The DPCs contents in nano-DPCs were measured by HPLC system, as mentioned above.

### 2.2. Animals

All the mice used in this study were purchased from Japan SLC, Inc. (Shizuoka, Japan). The mice were individually housed in plastic cages under a specific pathogen-free condition and maintained at 24 °C in a 12 h light-dark cycle. The mice were fed a normal commercial chow for 7 d to acclimate them to their environment, after which they were subsequently randomly divided into experimental groups for each experiment. All experimental animal protocols were approved by the animal experiment committee at University of the Ryukyus, Okinawa Japan, and the experiments were performed according to the ethical guidelines of the university for animal experiments.

To elucidate the anti-obesity effects of DPCs and its augmentation by nanoparticulation with PLGA, twenty-four 4-week-old male C57BL/6 mice were used in this study. Following 1-week acclimation, mice were randomly divided into the following four groups (*n* = 6 for each group): control, regular dose DPC (regular dose), low dose DPC (low dose), and low dose nanoparticulated DPC (nano-DPC) groups (Table 1). Experimental diets and DPC doses were optimized according to our previous study [14]. The experimental HFD containing 20% fat was prepared as the AIN-76 formulation and its composition is summarized in Supplementary Table S1. Approximately 39% of the total calories in HFD were derived from fat. Table 1 summarizes the administration procedures of DPCs in this experiment. The animals fed HFD were supplemented with DPCs either by diet or gavage. The control group was fed with only HFD without any supplementation of DPCs. The administration of DPCs to regular and low dose groups was by diet while to the nano-DPC group the administration was by gavage. In the case of supplementation by diet, DPCs was added at the expense of sucrose. In the nano-DPC group, nano-DPCs suspended in water were orally administered by gavage to mice (2 times/week). All groups, except the nano-DPC group, received the vehicle PLGA nanoparticles by gavage (2 times/week). All mice were given ad libitum access to the experimental diet and water for 10 weeks. Feces were collected for 3 days at the end of the feeding period and lyophilized. The mice were starved for 12 h before euthanasia under anesthesia by exsanguination from the heart. The liver and WAT were immediately excised, and the sera were prepared from the blood. Part of the excised epididymal WAT was fixed in 10% neutral formalin solution, and the remaining tissue and sera were frozen in liquid nitrogen and stored at −80 °C until use.

Eighteen male 6-week-old ICR mice were used to examine the effect of PLGA nanoparticle encapsulation on bioavailability of oral DPCs in this study. After giving them 1 week to acclimate to their environment, the mice were randomly divided into two groups (*n* = 9 for each group). The mice were starved for 12 h and then orally administered with purified DPC suspension with vehicle PLGA nanoparticle or nano-DPCs (DPC dosage 4 mg/kg body weight) by gavage. After treatment for 24 h, the mice were euthanized under anesthesia by exsanguination from the heart. Epididymal WAT was immediately excised, frozen in liquid nitrogen, and stored at −80 °C until use.

### 2.3. Lipid Concentrations and Biochemical Parameters in the Serum, Liver, and Feces

Serum levels of total cholesterol (TC), triglycerides (TG), and glucose and the aminotransferase activities as an indicator of hepatopathy were determined using a commercial enzymatic kit (Wako Pure Chemical Industries, Ltd., Osaka, Japan). Serum leptin levels were measured using enzyme-linked immunosorbent assay kits (Morinaga Institute Biological Science, Inc., Kanagawa, Japan). Lipids in the liver and feces were extracted using a previously described method [17], and their concentrations were determined using the commercial kits described above.

### 2.4. Adipocyte Size

Fixed epididymal WAT was embedded in paraffin and stained with hematoxylin and eosin to measure cell size using a microscope, according to previously described procedures [18]. The stained section was viewed at 10× magnification and photographed using a digital camera (Olympus BX41, Armonk, NY, USA). Analyses of the adipocyte area of at least 100 adipocytes per section were conducted using Adiposoft software plugin for ImageJ (https://imagej.nih.gov/ij/; version 1.16).

### 2.5. Quantitative Real-Time Polymerase Chain Reaction

Total RNA was extracted from epididymal WAT using a TRIzol reagent and PureLink RNA mini kit (Thermo Fisher Scientific, Waltham, MA, USA). First-strand cDNA was synthesized using 2 µg total RNA as a template. For quantitative real-time polymerase chain reaction, the primers and probe sets for acetyl-coenzyme A (CoA) carboxylase 1 (ACC1, Mm.PT.58.12492865), β-actin (ACTB, Mm.PT.58.33257376.gs), CCAAT-enhancer-binding protein 1 (C/EBPα, Mm.PT.58.30061639.g), carnitine palmitoyltransferase 1A (CPT1a, Mm.PT.58.10147164), fatty acid binding protein 4 (FABP4, Mm.PT.58.43866459), fatty acid synthase (FASN, Mm.PT.58.14276063), glyceraldehyde-3-phosphate dehydrogenase (GAPDH, Mm.PT.39a.1), hormone-sensitive lipase (LIPE, Mm.PT.58.6342082), lipoprotein lipase (LPL, Mm.PT.58.46006099), peroxisomal acyl-coenzyme A oxidase 1 (ACOX1, Mm.PT.58.50503784), peroxisome proliferator-activated receptor (PPAR) α (PPARα, Mm.PT.58.9374886), PPARγ (Mm.PT.58.31161924), PPARγ coactivator 1α (PGC1a, Mm.PT.58.16192665), stearoyl-CoA desaturase-1 (SCD1, Mm.PT.58.8351960), glucose transporter type 4 (GLUT4, Mm.PT.58.9683859), sterol regulatory element-binding protein 1 (SREBF1, Mm.PT.58.8508227), uncoupling protein (UCP) 1, (UCP1, Mm.PT.58.7088262), UCP2 (Mm.PT.58.11226903), and UCP3 (Mm.PT.58. 9090376) were purchased from Integrated DNA Technologies, Inc. (Coralville, IA, USA). To measure the relative abundance of target transcripts, amplifications were performed using PrimeTime Gene Expression Master Mix (Integrated DNA Technologies, Inc.) with the StepOne Real-Time PCR System (Thermo Fisher Scientific, Waltham, MA, USA), and the amounts of the target transcripts were normalized to those of ACTB and GAPDH. 

### 2.6. Ultraperformance Liquid Chromatography Analysis of DPCs Concentration in Epididymal WAT

One hundred milligrams of excised epididymal WAT used in experiment two were homogenized in chloroform/methanol (2/1) with 50 ng visnadine (Sigma-Aldrich, St. Louis, MO, USA) as an internal standard. After adding distilled water to the homogenate and centrifuging, the chloroform layer was collected and evaporated. The extracted lipid was filtered and subjected to liquid chromatography-mass spectrometry (LC-MS) analysis. The chromatographic analysis was conducted on a Waters Acquity Ultraperformance liquid chromatography (UPLC) H-Class system coupled to a Waters Xevo TQD mass spectrometer (Waters, Milford, MA, USA). In the LC system, a 10 × 2.1 mm Titan C18 UHPLC column (particle size: 1.9 µm; Sigma-Aldrich, St. Louis, MO, USA) was used at 40 °C, and the mobile phase was 65% acetonitrile with 0.1% formic acid at a flow rate 0.4 mL/min. The operating parameters for MS were as follows: capillary voltage, 3.00 kV; cone voltage, 55 V; source temperature, 150 °C; desolvation temperature, 500 °C; desolvation gas flow, 1000 L/h; and cone gas, 150 L/h. Quantification was conducted in positive electrospray ionization and multiple reaction monitoring (MRM) modes. MRM transitions were *m/z* 409.2 → 309.2 for peucedanocoumarin III (PCIII) and pteryxin (PTX) and *m/z* 411.2 → 351.1 for visnadine as the internal standard. Quantitative data analyses were conducted using the Waters MassLynx with TargetLynx application managers (Waters, Milford, MA, USA).

### 2.7. Statistical Analyses

Data are expressed as the mean ± standard error of the mean (SEM). The statistical significance of the difference between the two experimental groups was determined using the Student’s *t*-test. To determine the significance of the differences among the means for more than three groups, the data were analyzed using one-way analysis of variance, and the differences among the mean values were subsequently inspected using the Tukey’s honestly significant difference test. The level of significance was set to *p* < 0.05.

## 3. Results

### 3.1. DPC Content in Purified DPCs and Nano-DPCs

In this study, we purified DPCs from PJT. HPLC analyses showed that purified DPC extract has two major peaks, PCIII (41.7%, *v/v*) and PTX (57.3%, *v/v*) (Figure S1). Nano-DPCs were incorporated with 12.0% ± 0.2% of DPCs, PCIII 5.04%, and PTX 6.96%.

### 3.2. Effect of Dietary DPCs and Nano-DPCs Administration on Growth Parameters

The mice in the regular and low dose groups were fed the experimental HFD containing DPCs at 0.069% and 0.00069%, respectively, which meant that mice in the regular dose DPC group took a total of 146 ± 1 mg DPCs, while the mice in low dose DPC group received only 1% of that amount (Table 1). As a result, the total intake of DPCs in the nano-DPC group mice which were fed the control HFD but also administered nano-DPCs twice a week was comparable to that in the low dose DPC group mice (Table 1).

**Figure 2:**
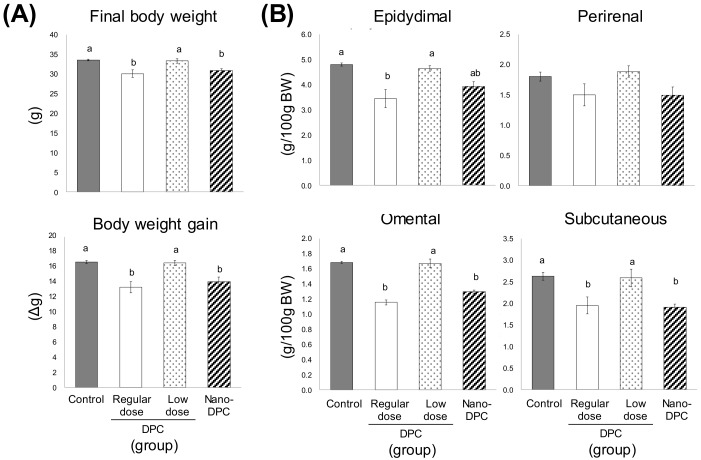
Effects of dietary dihydropyranocoumarins (DPCs) and DPC-encapsulated polylactic-co-glycolic acid nanoparticles (nano-DPCs) on growth parameters. (**A**) Final body weight and body weight gain and (**B**) relative weights of white adipose tissues. Notes: Each value represents the mean ± SEM for six mice. Different letters indicate significant differences among each experimental group using Tukey’s honestly significant difference test (*p* < 0.05).

The total food and energy intake, and the fecal TG content over the final three experimental days were largely comparable among all experimental groups (Table S2). However, significant decreases in the final body weight and body weight gain were observed in the regular dose and nano-DPC groups as compared with the control and low dose groups (Figure 2A). The relative weights of epididymal, omental, and subcutaneous WAT in the regular dose group were significantly lower than those in the control and low dose groups (Figure 2B). Similar results were observed in the nano-DPC group, but their epididymal WAT showed a tendency to decrease (Figure 2B). The weight of relative perirenal WAT in mice of the regular dose and nano-DPC groups also showed a tendency to decrease as compared with that of the control and low dose groups (Figure 2B).

### 3.3. Effect of Dietary DPCs and Nano-DPCs Administration on Serum and Hepatic Parameters

**Figure 3:**
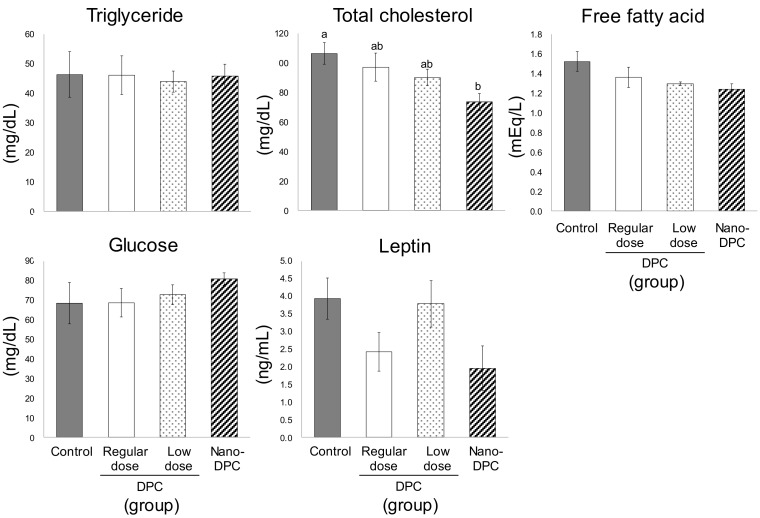
Effects of dietary dihydropyranocoumarins (DPCs) and DPC-encapsulated polylactic-co-glycolic acid nanoparticles (nano-DPCs) on serum parameters. Each value represents the mean ± SEM for six mice. Different letters indicate significant differences among each experimental group using Tukey’s honestly significant difference test (*p* < 0.05).

**Figure 4:**
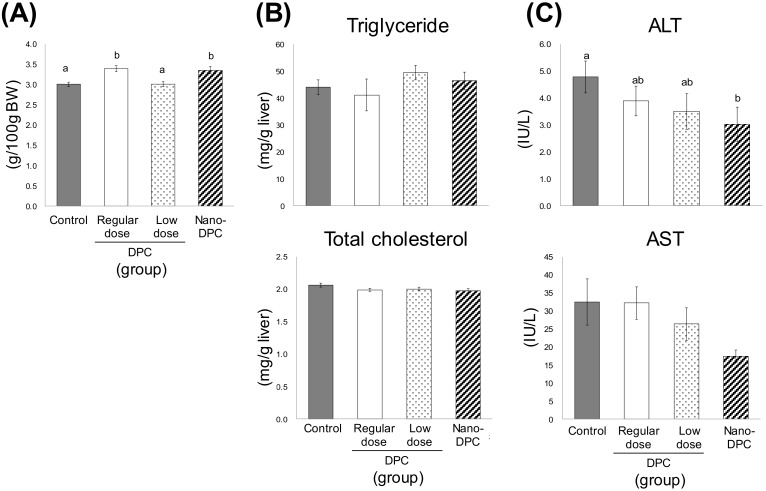
Effects of dietary dihydropyranocoumarins (DPCs) and DPC-encapsulated polylactic-co-glycolic acid nanoparticles (nano-DPCs) on hepatic parameters. (**A**) Relative liver weight, (**B**) hepatic lipid contains, and (**C**) alanine transaminase activities. Notes: ALT, alanine aminotransferase and AST, aspartate aminotransferase. Each value represents the mean ± SEM for six mice. Different letters indicate significant differences among each experimental group using Tukey’s honestly significant difference test (*p* < 0.05).

As shown in Figure 3, serum levels of TG, free fatty acid, and glucose were largely comparable among all experimental groups; however, serum TC level in mice of the nano-DPC group significantly decreased as compared with that of the control group. Serum leptin levels in regular dose and nano-DPC groups also tended to decrease as compared with those in the control and low dose groups. Although the raw weight of the liver was largely comparable among all experimental groups (data not shown), its relative weight in mice of regular dose and nano-DPC groups was significantly lower than that of the control and low dose groups (Figure 4A). Oral intake of DPCs by diet and oral injection of nano-DPCs by gavage had no effect on hepatic lipid levels (Figure 4B). For the hepatopathy indicators, serum alanine aminotransferase (ALT) activity in the nano-DPC group mice significantly decreased, and a decreasing tendency in aspartate aminotransferase (AST) activity was also observed in those mice as compared with those in the control group mice (Figure 4C).

### 3.4. Effects of DPCs and Nano-DPCs on Lipid Accumulation in Epididymal Adipose Tissues

**Figure 5:**
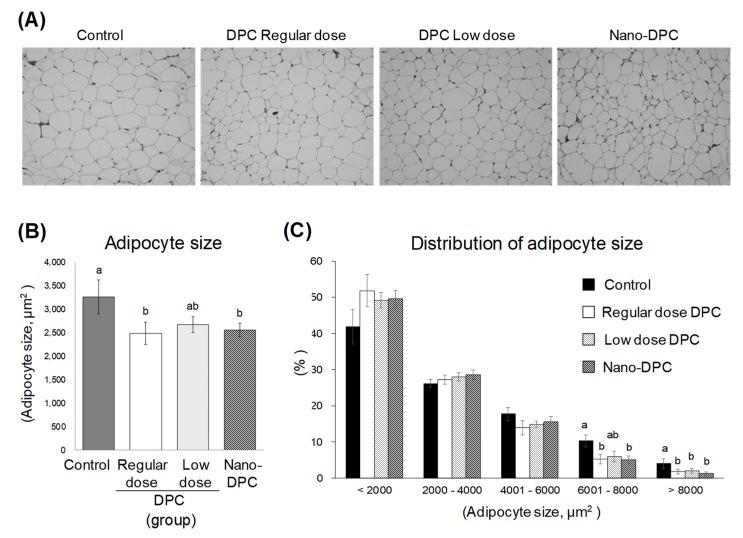
Effects of dietary dihydropyranocoumarins (DPCs) and DPC-encapsulated polylactic-co-glycolic acid nanoparticles (nano-DPCs) on adipocyte size in epididymal white adipose tissues. (**A**) Histological images of epididymal WAT, (**B**) mean of adipocytes size, and (**C**) distribution of adipocyte size. Notes: Each value represents the mean ± SEM for six mice. Different letters indicate significant differences among each experimental group using Tukey’s honestly significant difference test (*p* < 0.05).

Dietary intake of DPCs significantly affected the size of adipocyte in epididymal WAT (Figure 5A). Mice in the regular dose and the nano-DPC groups had significantly smaller adipocytes than the control group mice (Figure 5B). In the regular dose and nano-DPC groups, the proportion of large adipocytes (>6000 μm^2^) significantly decreased and that of small adipocytes (<2000 μm^2^) tended to increase as compared with those in the control group (Figure 5C). Although the relative weights of epididymal WAT were largely comparable between the control and the low dose group mice, a tendency for the mean adipocyte size to decrease was observed in the low dose group (Figure 5B). The percentage of large adipocytes (>8000 μm^2^) in the low dose group mice was significantly lower than that in the control group mice (Figure 5C).

### 3.5. Effect of Dietary DPCs and Nano-DPCs Administration on Lipid Metabolism–Related Gene Expressions in Epididymal WAT

**Figure 6:**
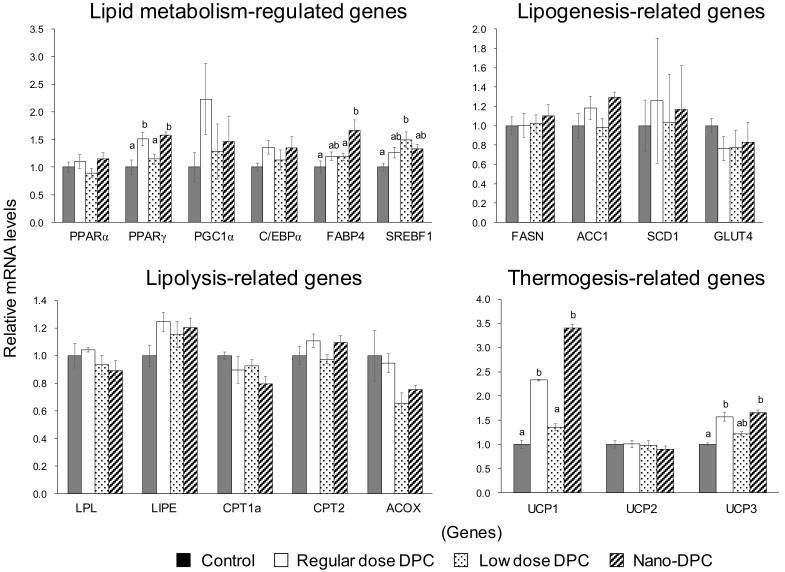
Effects of dietary dihydropyranocoumarins (DPCs) and DPC-encapsulated polylactic-co-glycolic acid nanoparticles (nano-DPCs) on lipid metabolism-related gene expression in epididymal white adipose tissue. Notes: Each value represents the mean ± SEM from at least three independent experiments. Different letters indicate significant differences among each experimental group using Tukey’s honestly significant difference test (*p* < 0.05).

To understand the underlying mechanisms by which DPCs and nano-DPCs affect epididymal WAT on a genetic level, we evaluated the mRNA levels of lipid metabolism-related genes in this tissue (Figure 6). The relative mRNA levels of PPARγ and FABP4 in mice of the regular dose and the nano-DPC groups were significantly higher than those of the control group. Increasing tendencies were also observed in mRNA levels of PGC1α and C/EBPα in the mice fed DPCs. In mice of the low dose group, mRNA levels of SREBF1 significantly increased as compared with that of the control group. Significant differences were not observed in relative mRNA levels of lipogenesis- and lipolysis-related genes, and dietary intake of DPCs and oral administration of nano-DPCs significantly increased thermogenesis-related gene expressions in WAT. The relative mRNA levels of UCP1 in WAT in mice of the regular dose and nano-DPC groups significantly increased by 2.5- to 3.5-fold as compared with those of the control and low dose groups. Gene expression of UCP3 in mice of the regular dose and nano-DPC groups also significantly increased as compared with that in the control group mice, but not compared with that in the low dose group mice.

### 3.6. Effect of PLGA Nanoparticulation on the Concentration of DPCs in WAT

**Figure 7:**
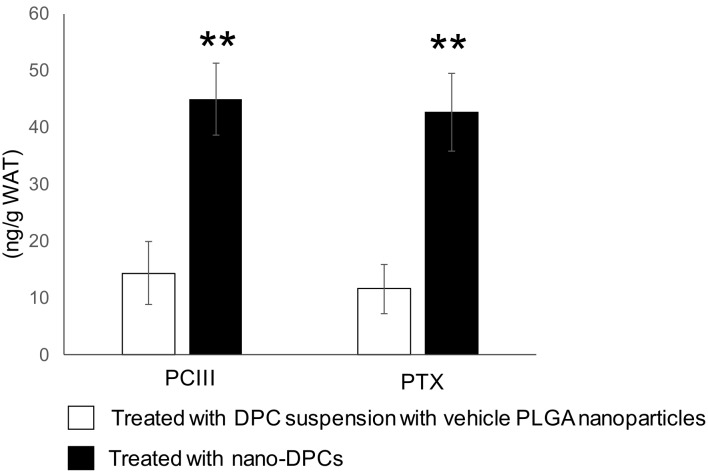
Concentration of dihydropyranocoumarins (DPCs) in epididymal white adipose tissue (WAT) collected 24 h after oral administration of DPCs or polylactic-co-glycolic acid (PLGA) nanoparticles with DPCs (nano-DPCs). Notes: Each value represents the mean ± SEM for 9 mice. The asterisk shows significant differences as compared with the mice treated with DPC suspension with vehicle PLGA nanoparticles using the Student’s *t*-test (** *p* < 0.01).

To understand the effect of PLGA nanoparticulation of DPCs on WAT, we compared the DPC content in WAT after a single oral administration of DPCs and nano-DPCs by gavage. The PCIII content in WAT in mice administered with nano-DPCs showed an approximately three-fold higher level than when using a DPC suspension with vehicle PLGA nanoparticles (Figure 7). Similar results were also observed in the PTX contents in WAT.

## 4. Discussion

In this study, we investigated the effects of DPCs, such as PCIII and PTX isolated from PJT, on the development of obesity in HFD-fed mice. Although the results of our previous study did not remove the possibility that other ingredients in DPC concentrate (purity ~60%) have anti-obesity effects [14], the results of this study suggested that dietary intake of pure DPCs prevents fat accumulation in WAT, and that DPCs are a significant factor in the anti-obesity effects of PJT. In addition, the results suggested that DPCs’ anti-obesity properties are caused by an increase in energy expenditures resulting from increased UCPs, and that PLGA-based nanoparticulate system is a powerful tool to enhance DPC activities.

Adipose tissue is the most flexible endocrine organ that can expand and reconstruct itself throughout life. Adipose tissue can expand either by hypertrophy or hyperplasia [19]. On the one hand, hypertrophy of adipocytes can increase hypoxia and mechanical stress to neighboring cells and the extracellular matrix, resulting in decreased adipose tissue function, which contributes to the early onset of metabolic disease, and persistently elevated levels of nutrients in the blood, which cause toxic lipid deposits in other tissues, such as muscle and the liver [20,21]. On the other hand, hyperplastic growth is considered to be a healthy and adaptive mechanism by which to maintain proper vascularization, responses to anti-inflammatory hormone adiponectin, and insulin-sensitizing and other metabolism-modulatory adipokines [20,22]. Indeed, although treatment with thiazolidinediones, an insulin-sensitizing drug, leads to enhancement of overall adipose tissue growth, it induces the conversion of hypertrophic into hyperplastic adipose tissue, which results in a greater number of small adipocytes and a significant decrease in large adipocytes [23]. In hyperplasia of adipocytes, mature adipocytes are generated from preadipocytes during adipogenesis, which is principally regulated by C/EBPα and FABP4 [24,25]; therefore, it is known that hypertrophic obesity is also more strongly associated with insulin resistance and metabolic complications than that of hyperplastic obesity [26]. In this study, both gene expressions significantly increased or tended to increase in epididymal WAT of the mice fed HFD containing DPCs (Figure 6). Dietary intake of DPCs also led to decreases in the mean size of white adipocytes (Figure 5). One recent study has reported that dietary intake of suksdorfin, which has a structure similar to that of the DPCs used in this study, induces adipogenesis in WAT in obese, diabetic *KK-Ay* mice [27]. Thus, these results suggested that the dietary intake of DPCs can induce the conversion of hypertrophic into hyperplastic obesity in HFD-fed mice. It has also suggested that other ingredients in PJT can exert antilipogenic effects on WAT because our previous study observed significant decreases in mRNA levels of lipogenesis-related genes in the epididymal WAT of mice fed HFD containing DPC concentrates from PJT (DPC contents ~62.2%) [14]. In addition, it should be noted that these phenomena were observed in the low dose group, in which mice were fed 1% of the amount of DPCs fed to the regular dose group (Table 1).

As mentioned above, the dietary intake of DPCs and oral administration of nano-DPCs significantly decreased hypertrophic white adipocytes (Figure 5). However, significant decreases in the mass of epididymal WAT were observed in mice of regular dose and nano-DPC groups, but not in the low dose group mice (Figure 2B). This decreased WAT mass contained significant upregulated mRNA levels of thermogenesis-related genes, such as UCP1 and UCP3 (Figure 6). It is well known that UCPs increase proton leakage across the mitochondrial inner membrane, and thereby dissipates the proton motive force as heat instead of synthesizing ATP [28]. Adipocytes are broadly divided into white adipocytes and brown adipocytes. Brown adipocytes are characterized by multilocular lipid droplets and have thermogenic properties mainly through the mitochondrial UCP1 in brown adipose tissues, whereas white adipocytes store neutral fat as energy in WAT [29]. It has been reported that adaptive stimuli, such as cold exposure and adrenergic stimulation, can induce browning, which converts white adipocytes into brown-like adipocytes (recently called “beige” adipocytes) in WAT [30]. These beige adipocytes resemble classical brown adipocytes with high UCP1 expression; therefore, it is suggested that their generation increases energy expenditure and can prevent obesity. [31,32,33]. At the molecular level, WAT browning is regulated by multiple factors and signaling pathways. PGC-1α is known as a cold-induced interacting partner of PPARγ in adipocyte browning [34]. Consistent with our findings on the upregulations of PPARγ, PGC-1α, and UCP1 expression in WAT (Figure 6), we observed that the dietary intake of DPCs or oral treatment with nano-DPCs enhanced energy expenditure and caused significant decreases in epididymal WAT mass in regular dose and nano-DPC group mice (Figure 2). In addition, these results suggested that the biological activities of DPCs are factors in the conversion of white into beige adipocytes.

PLGA nanoparticles have markedly improved the bioavailability of several compounds such as curcumin after oral administration [8,35,36]. One recent study has demonstrated that incorporation into PLGA nanoparticles markedly augment the therapeutic effects of γ-oryzanol on glucose and lipid metabolism in obese diabetic mice [37]. In this study, we observed that treatment with a small amount of DPC (1% of DPC amount in regular dose) had no effect on WAT weight but had significant anti-obesity effects on mice when the same amount of DPCs encapsulated into PLGA nanoparticles (Figure 2 and Figure 5). Moreover, PLGA nanoparticle encapsulation significantly increased the DPC concentration in WAT 24 h after oral administration (Figure 7). These results might demonstrate the huge potential for PLGA nanoparticles to act as carriers for the oral delivery of DPCs. Moreover, significant decreases in serum levels of cholesterol and hepatopathy indicators were observed in mice treated with DPCs encapsulated into PLGA, but not in those treated with DPCs alone (Figure 3 and Figure 4). It has been reported that the compound encapsulated into PLGA nanoparticles is distributed mainly in the liver and intestine in mice over a longer period time than the non-encapsulated compound after oral administration [37]. Therefore, it is suggested that PLGA nanoparticle encapsulation expands the therapeutic potential of DPCs to prevent the development of obesity and its related diseases, although further studies are needed to confirm this effect.

## 5. Conclusions

The present study demonstrated that DPCs, such as PCIII and PTX contribute to the beneficial effects of PJT on the development of obesity by converting hypertrophic to hyperplastic obesity and enhancing energy expenditures. Although additional studies are needed to elucidate the underlying details of the mechanisms by which these activities have an effect on obesity, our study suggests the great potential for PLGA nanoparticle encapsulation to be carriers of the oral delivery of DPCs in the development of natural and safe anti-obesity agents.

## Figures and Tables

**Table 1:** Administration procedure of dihydropyranocoumarins (DPC) and its total dosages in this study.

	Experimental Group			
	Control	Regular Dose DPC	Low Dose DPC	Nano-DPC
Total DPC dosage (mg) ^§^				
by diet	0	146 ± 1	147 ± 0	0
by gavage	0	0	0	1.48 ± 0
Oral administration by gavage (µg/injection) ^†^				
Nano-DPC	0	0	0	615 ^‡^
Vehicle PLGA nanoparticles	541	541	541	0

^§^ Data are shown as the means ± SEM. ^†^ nano-DPC, DPC-encapsulated polylactic-co-glycolic acid (PLGA) nanoparticles. Nano-DPC and vehicle PLGA nanoparticles were orally administered twice a week. ^‡^ 615 µg nano-DPC contains 73.8 µg DPCs.

## Supplementary Materials

The following are available online at https://www.mdpi.com/2072-6643/11/12/3053/s1, Table S1: Composition of experimental diets used in this study, Table S2: Total food and energy intake, and fecal lipid excretion, Figure S1: HPLC chromatogram of DPC concentrate.
