# Supplementary material for: Dihydropyranocoumarins Exerted Anti-Obesity Activity In Vivo and its Activity Was Enhanced by Nanoparticulation with Polylactic-Co-Glycolic Acid
Source: Nutrients. 2019 Dec 13;11(12):3053. doi: 10.3390/nu11123053 (PMC6949991; doi:10.3390/nu11123053)
Supplement: Supplementary file 1 [file nutrients-11-03053-s001.pdf]

Supplementary Table S1. Composition of experimental diets used in this study

| | Experimental group | | | |
|---|---|---|---|---|
| | Control | Regular dose DPC | Low dose DPC | Low dose nano-DPC |
| Composition of experimental diet (g/100g) | | | | |
| Casein | 20.0 | 20.0 | 20.0 | 20.0 |
| β-corn starch | 15.0 | 15.0 | 15.0 | 15.0 |
| Cellulose | 5.00 | 5.00 | 5.00 | 5.00 |
| AIN-76 mineral mix | 3.50 | 3.50 | 3.50 | 3.50 |
| AIN-76 vitamin mix | 1.00 | 1.00 | 1.00 | 1.00 |
| DL-methionine | 0.300 | 0.300 | 0.300 | 0.300 |
| Choline bitartrate | 0.200 | 0.200 | 0.200 | 0.200 |
| Corn oil | 20.0 | 20.0 | 20.0 | 20.0 |
| Purified DPC | - | 0.069 | 0.00069 | - |
| Sucrose | to make 100 | | | |

Supplementary Table S2. Total food and energy intake, and fecal lipid excretion

| | Experimental group | | | |
|---|---|---|---|---|
| | Control | Regular dose DPC | Low dose DPC | Low dose nano-DPC |
| Total food intake (g) | 199 ± 1 | 197 ± 1 | 198 ± 1 | 199 ± 1 |
| Total energy intake (kcal) | 914 ± 5 | 904 ± 8 | 912 ± 3 | 914 ± 4 |
| Fecal lipid excretion (mg/3days) | 395 ± 32 | 384 ± 16 | 359 ± 23 | 362 ± 11 |

Data are shown as the means ± SEM.

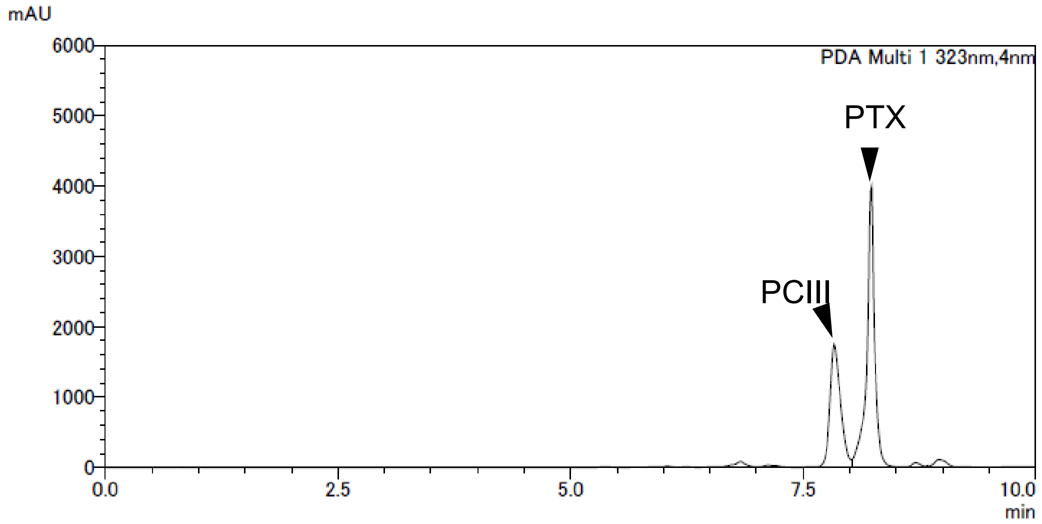


Supplementary Fig. 1 HPLC chromatogram of DPC concentrate
Notes: PCIII: peucedanocoumain III, PTX: pteryxin.
